# Supplementary material for: Plane photoacoustic wave generation in liquid water using irradiation of terahertz pulses
Source: Sci Rep. 2020 Oct 28;10:18537. doi: 10.1038/s41598-020-75337-6 (PMC7595166; doi:10.1038/s41598-020-75337-6)
Supplement: Supplementary file 3 — Supplementary Information. [file 41598_2020_75337_MOESM3_ESM.pdf]

Supplementary Materials for

Plane photoacoustic wave generation in liquid water using irradiation of terahertz pulses

Masaaki Tsubouchi,[1]* Hiromichi Hoshina,[2] Masaya Nagai,[3] Goro Isoyama[4]

*Corresponding author. Email: tsubouchi.masaaki@qst.go.jp

This file includes:

Pulse width measurement of the THz micropulse
Single THz-pulse pick-up

Figure S1. THz-FEL spectra and absorption spectrum of liquid water.
Figure S2. THz micropulse intensity profile in time-domain.
Figure S3. Calculated power reflectivity of the THz light from the photoexcited GaAs wafer.

Other Supplementary Material for this manuscript includes the following:

Video S1. Time evolution of the photoacoustic waves induced by the THz pulse train during 1 μs.
Video S2. Time evolution of the photoacoustic waves induced by the pick-up single THz pulse during 4.5 μs.

Supplementary Materials

Pulse width measurement of the THz micropulse

We evaluated the temporal profile of the THz micropulses by an electro-optic sampling method[1,2] using a Ti:sapphire laser (Tsunami, Spectra-Physics) with a repetition rate of 81 MHz and a pulse duration of 100 fs which was synchronized to the third harmonic of the micropulse repetition (27 MHz) in the THz-FEL with a jitter of < 2 ps. We used a (110)-oriented 0.27 mm thick GaP crystal as the electro-optic crystal and a polarimeter based on detectors with the bandwidth of 200 MHz. Figure S2 shows a temporal intensity profiles of the single THz micropulse. We evaluated the typical value of the pulse width of the single micropulse as 1.7 ps.

Single THz-pulse pick-up

Figure 2(B) shows the single THz-micropulse pick-up from the pulse train by the plasma mirror with nanosecond gating.[3,4] We employed a GaAs wafer irradiated by an intense femtosecond Ti:sapphire laser pulse as the nanosecond plasma mirror. The GaAs is transparent to THz light and has a Brewster angle of 75° for the p-polarized THz light. When the GaAs wafer is irradiated by the near-IR light, a dense electron plasma is generated on the surface with a minority carrier lifetime of less than 10 ns.[5] Then, the GaAs plasma mirror can pick up only a single micropulse from the pulse train with a time interval of 36.9 ns.

In our experiment, the optical pump light was provided by the Ti:sapphire regenerative amplifier with the wavelength of 800 nm, the pulse duration of 100 fs, the pulse energy of 500 μJ. This pump light irradiated the GaAs surface with a spot size of 1 cm^2, and generated the thin dense carrier layer with the density of 10^{19} cm^{-3} which was estimated by the penetration depth of the optical light into the GaAs, 1 μm, corresponding to the absorption coefficient of 10^4 cm^{-1} at the wavelength of 800 nm. In this estimation, we also included the Fresnel loss of the pump light at the GaAs surface.

Figure S3 shows the calculated power reflectivity of the THz light from the GaAs wafer. In this calculation, we assumed the THz light with the incident angle of 75° and p-polarization. On the surface of GaAs, the carrier was formed with the density of 10^{16} ~ 10^{19} cm^{-3} and with the thickness of 1 μm. The calculation was performed with the parameters

obtained in the previous work by one of the authors.[6] With the carrier density larger than 10^{18} cm^{-3}, the THz reflectivity drastically increases to 90%. In our present experiment, the carrier density on the GaAs surface was estimated to be 10^{19} cm^{-3}. Therefore, the high reflectivity for the p-polarized THz light and the effective single micropulse pick-up were expected. However, in the actual experiment, due to the imperfect Brewster condition, the small reflectivity for the p-polarized THz light is still remained without the carrier formation on the GaAs. This is the reason why the residual micropulses appear in Fig. 2(B). To minimize the residual micropulses, the electron beam injection was stopped just after the pick-up pulse.

We also have to consider how the optical light absorption on the GaAs surface changes the reflectivity and absorbance of the THz light. In our present experiment, the low-density residual carriers with long lifetime (> 1 ms) is remained before and after the pump pulse irradiation because we use the optical pump light with the 1 kHz repetition rate. But Fig. S3 indicates that the residual carrier with the density lower than 10^{17} cm^{-3} does not enhance the reflectivity with the p-polarized THz light at the frequency from 3 to 7 THz.

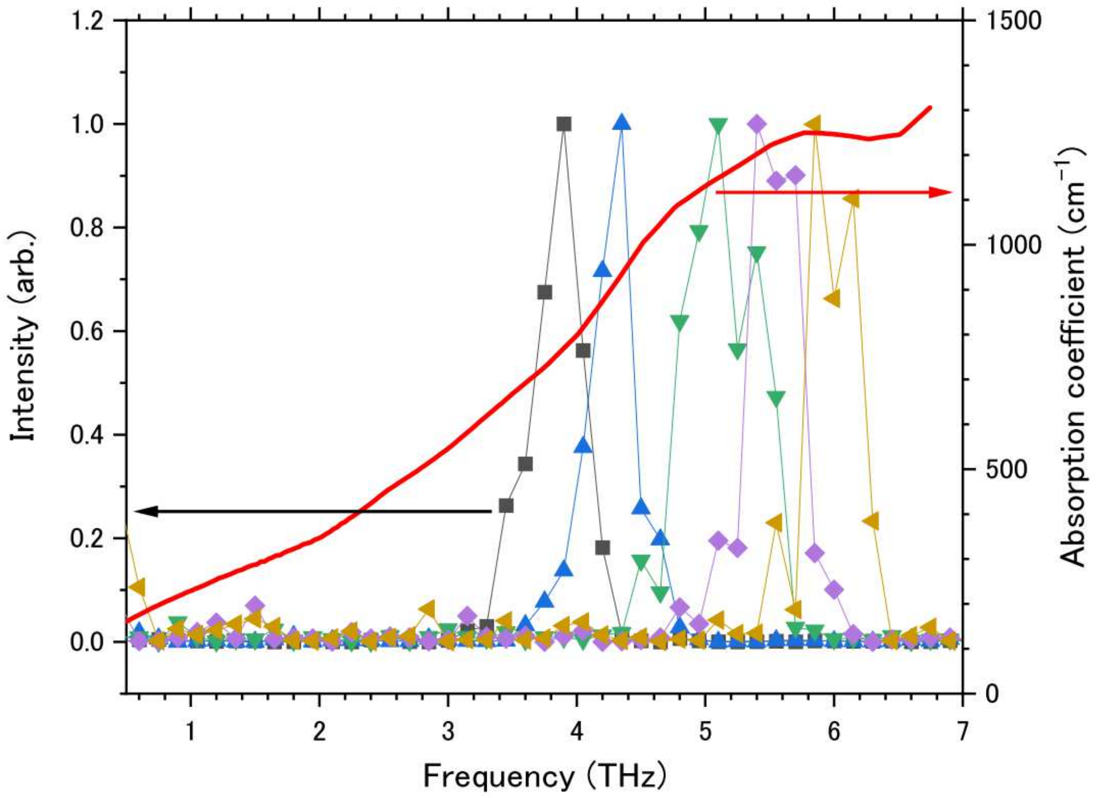

Figure S1. THz-FEL spectra and absorption spectrum of liquid water. The dot lines show the THz-FEL spectra observed while scanning the wiggler magnet gap from 36 to 45 mm. The absorption spectrum of liquid water at a room temperature is also shown as the red solid line.

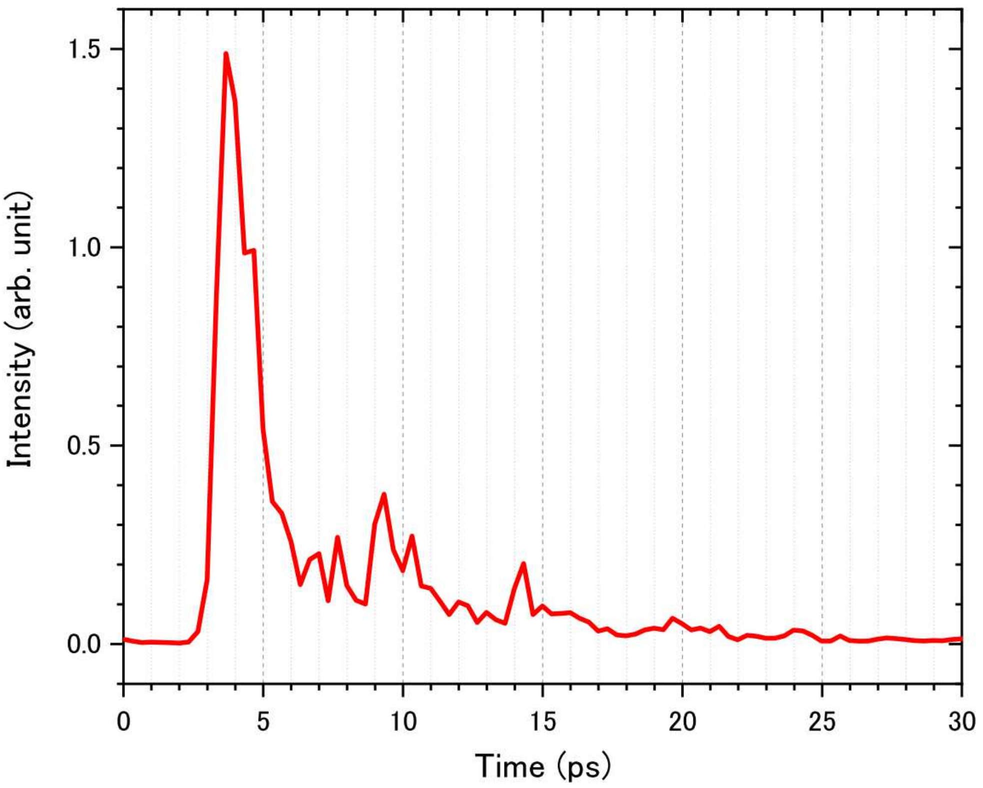

Figure S2. THz micropulse intensity profile in time-domain.

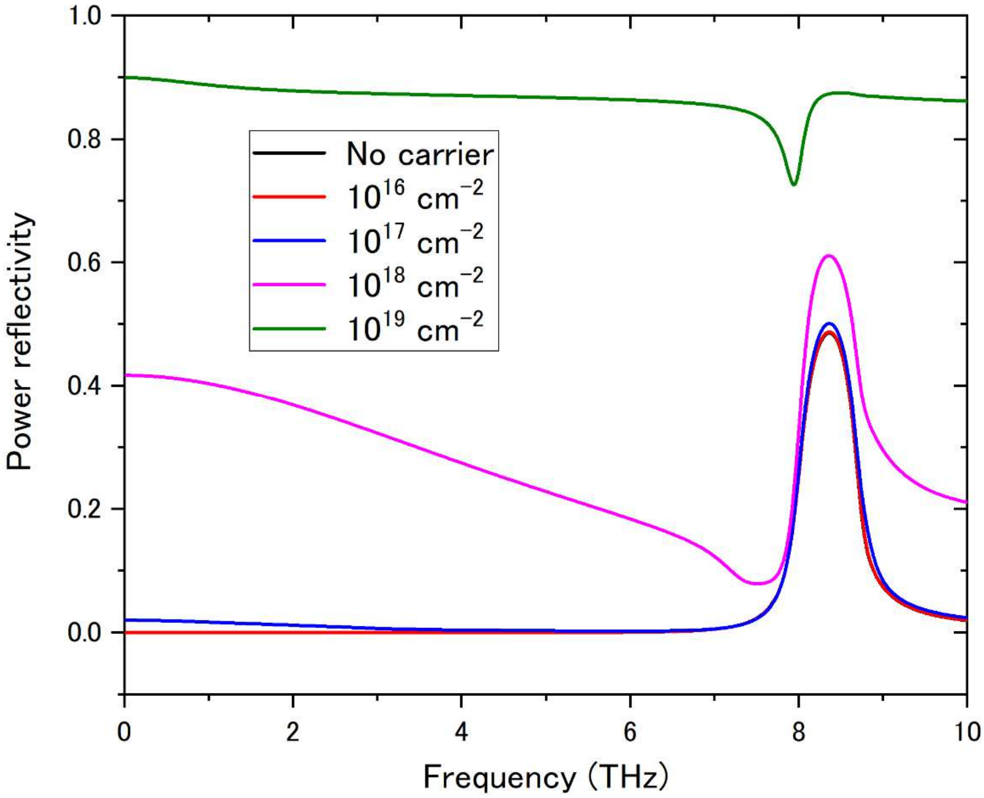

Figure S3. Calculated power reflectivity of the THz light from the photoexcited GaAs wafer. The THz light incidents on the photoexcited GaAs with the incident angle of 75° and p-polarization. On the surface of GaAs, the carrier is formed with the density of 10^{16} ~ 10^{19} cm^{-3} and with the thickness of 1 μm.

Supplementary Movie Captions

Video S1. Time evolution of the photoacoustic waves induced by the THz pulse train during 1 μs. This is the original sequential images to obtain the amplitude map shown in Fig. 3(b). The images were captured with a time gate of 10 ns at a time interval of 10 ns.

Video S2. Time evolution of the photoacoustic waves induced by the pick-up single THz pulse during 4.5 μs. The images were captured with a time gate of 10 ns at a time interval of 15 ns.

References

1 Nahata, A., Weling, A. S. & Heinz, T. F. A wideband coherent terahertz spectroscopy system using optical rectification and electro-optic sampling. *Appl. Phys. Lett.* **69**, 2321-2323 (1996).

2 Wu, Q. & Zhang, X. C. Design and characterization of traveling-wave electrooptic terahertz sensors. *IEEE J. Sel. Top. Quantum Electron.* **2**, 693-700 (1996).

3 Knippels, G. M. H. & van der Meer, A. F. G. FEL diagnostics and user control. *Nuclear Instruments and Methods in Physics Research Section B: Beam Interactions with Materials and Atoms* **144**, 32-39, doi:https://doi.org/10.1016/S0168-583X(98)00347-4 (1998).

4 Wang, X., Nakajima, T., Zen, H., Kii, T. & Ohgaki, H. Damage threshold and focusability of mid-infrared free-electron laser pulses gated by a plasma mirror with nanosecond switching pulses. *Appl. Phys. Lett.* **103**, 191105, doi:10.1063/1.4828995 (2013).

5 Sze, S. M. & Ng, K. K. *Physics of Semiconductor Devices*. (Wiley, 2006).

6 Yamashita, G. *et al.* Sensitive monitoring of photocarrier densities in the active layer of a photovoltaic device with time-resolved terahertz reflection spectroscopy. *Appl. Phys. Lett.* **110**, 071108, doi:10.1063/1.4975631 (2017).
